# Oxidative stress and autophagy-mediated immune patterns and tumor microenvironment infiltration characterization in gastric cancer

**DOI:** 10.18632/aging.205194

**Published:** 2023-11-09

**Authors:** Jifeng Liu, Biao Zhang, Yunshu Zhang, Huahui Zhao, Xu Chen, Lei Zhong, Dong Shang

**Affiliations:** 1 Department of General Surgery, The First Affiliated Hospital of Dalian Medical University, Dalian, China

**Keywords:** oxidative stress, autophagy, gastric cancer, molecular features, tumor microenvironment

## Abstract

Recent years have seen a sharp rise in the amount of research on the connection between oxidative stress, autophagy, and cancer cells. However, the significant functions of oxidative stress and autophagy-related genes (OARGs) in gastric cancer (GC) are yet to be investigated integrally. Therefore, it will be a new and promising concept to search for novel OARG-related biomarkers to predict the prognosis and treatment response of GC. First, we assessed changes in prognosis and tumor microenvironment (TME) characteristics across the various oxidative stress and autophagy-related modification patterns based on a detailed analysis of 17 OARGs with prognostic significance of 808 GC samples. We identified three distinct OARG alteration patterns which displayed unique biological characteristics and immune cell infiltration features. Using principal component analysis methods, the OARGscore was developed to evaluate the OARG modification patterns of certain tumors. The negative connection between OARGscore and immune cells was statistically significant. Increased survival, a higher incidence of mutations, and a better response to immunotherapy were all predicted to be related to patients’ high-OARGscore. In addition, the candidate chemotherapeutic drugs were predicted using the oncoPredict program. The low-OARGscore group was predicted to benefit more from Ribociclib, Alisertib, Niraparib, Epirubicin, Olaparib, and Axitinib, while patients in the high-OARGscore group were predicted to benefit more from Afatinib, Oxaliplatin, Paclitaxel, 5−Fluorouracil, Dabrafenib and Lapatinib. Our findings offer a specific method for predicting a patient’s prognosis and susceptibility to immunotherapy, as well as a promising insight of oxidative stress and autophagy in GC.

## INTRODUCTION

Among the most common forms of cancer, gastric cancer (GC) is a leading cause of cancer-related death worldwide, especially in East Asia [1, 2]. The pathological kind of more than 95% of cases of GC are of the stomach adenocarcinoma (STAD) [3]. The usual course of action for GC is radical resection followed by adjuvant therapy. Despite accepting normal treatment, the 5-year survival rate is just 50%, and 70% of patients with GC experience recurrence or metastasis within 5 years [4]. Sadly, the needs for earlier diagnosis and a longer survival duration are not met now by GC diagnosis and treatment. Therefore, research into novel biomarkers with higher predictive values is urgently needed to enhance GC prognostication.

Oxidative stress (OS) refers to the excessive accumulation of highly reactive molecules, such as reactive oxygen species (ROS) and reactive nitrogen species (RNS), in the body when subjected to various harmful stimuli, resulting in an imbalance of oxidative and antioxidant activity, leading to physiological and pathological responses from cells and tissues. Increased intracellular ROS concentrations can be caused by a number of conditions, including radiation, aging, viral illnesses, and heat stress. This can result in an intracellular OS response that can either protect or kill cells [5, 6]. According to prior studies, ROS has a significant role in all stages of cancer development, progression, and death [7, 8]. Additionally, several studies have demonstrated that OS can halt tumor growth and metastasis [9–11]. There is also evidence that GC may be reduced by reducing OS [12]. OS has also been linked to the tumor immune microenvironment (TME) and demonstrated to regulate the activity of immune cells in ovarian cancer [13]. Likewise, a link between OS-induced apoptosis and TME has been discovered in patients with gastric and esophageal cancers, which can influence patient prognosis [14].

A conserved intracellular breakdown process called autophagy uses lysosomes to break down cellular organelles, proteins, and invader germs to provide cells their essential building blocks and energy. It has a dual impact on tumor development and metastasis. In the early phases of carcinogenesis, for example, autophagy functions as a tumor-suppressing mechanism that restrains inflammation, preserves genomic stability, and protects against both chronic tissue harm and cellular damage [15–18]. Moreover, autophagy has diverse functions in various TME, and tumors can frequently alter the development and spread of tumors by controlling autophagy and, in turn, the immune response [19, 20]. It’s interesting to note that OS and ROS influence autophagy [21, 22]. Although the precise relationships between oxidative stress and autophagy have not been fully explored, it is known that OS may affect autophagy, typically increasing its induction [23–25]. At the same time, as far as we know, the OS and autophagy-related genes (OARGs) have not been uncovered in predicting clinical outcomes and therapeutic approaches in individuals of GC. Therefore, it is prospective to see the GC prognostic signature and developing better immunotherapy treatment options by employing OARG.

We began this study by looking at the genetic variants and expression characteristics for OARG in GC. In order to conduct a thorough investigation of OARG change patterns and TME characterization, we then selected genomic data of 808 GC cases. By cluster analysis based on OARGs with prognostic significance, the GC samples were split into three groups with considerably different prognoses and TME, showing that OARG modifications had a major influence on the progression of specific TME traits. In order to achieve accurate prediction of precisely individualized patients, we established the OARGscore for assessing the efficacy of immunotherapy and forecasting the prognosis of people with GC. At last, we also evaluated the correlation between OARGscore and commonly used chemotherapy drugs in order to better guide clinical medication.

## MATERIALS AND METHODS

### Data collection

The TCGA-STAD dataset containing 375 tumor samples and 32 normal samples, including their annotated clinical and gene expression data, were all obtained from The Cancer Genome Atlas (TCGA). 433 GC samples were obtained from the GSE84437 cohort of the Gene Expression Omnibus (GEO) [26]. Genes that correspond to multiple probes should all be selected for the average expression value of that gene. We normalized the matrix data and removed the batch effect using the “limma” and “sva” packages in R [27–29]. Besides, 1495 OS-related genes (Relevance score > 5) were obtained from the GeneCards database. GeneCards is a searchable, integrated database that offers thorough, user-friendly details on every human gene that has been annotated or predicted. A total of 222 autophagy-related genes were obtained from The Human Autophagy Database [30]. 96 OARGs were gained by taking the intersection of the two gene groups, which were then represented using a Venn diagram. Using univariate Cox regression [31], the TCGA and GEO cohorts were checked for OARGs with prognostic values (*P* < 0.05). At last, 17 OARGs of prognostic relevance were found.

### Sample collection

During the period spanning from 2023-06 to 2023-08, we meticulously procured a total of six sets of gastric cancer tissue samples along with an equivalent number of corresponding adjacent tissues. These specimens were meticulously obtained from the esteemed First Affiliated Hospital of Dalian Medical University. Notably, the origin of each of the aforementioned six pairs of human tissue samples can be traced back to six distinct patients who had undergone rigorous pathological scrutiny to confirm the presence of gastric cancer. It is imperative to highlight that each patient subjected themselves to standardized preoperative interventions, thereby excluding any prior history of chemotherapy or radiation therapy. The designated adjacent tissues, deliberately situated at a minimum distance of more than two centimeters from the epicenter of the neoplastic lesion, were meticulously selected. Subsequent to surgical excision, the harvested human tissue specimens were promptly subjected to cryopreservation within liquid nitrogen, effectively arresting the degradation of RNA molecules and ensuring their suitability for ensuing RNA isolation procedures. It is noteworthy to mention that the implementation of this research endeavor garnered the explicit endorsement and ethical oversight from the Ethics Committee of the First Affiliated Hospital of Dalian Medical University.

### Real-time quantitative PCR

Following the guidelines provided in the reagent specification, TRIzol reagent (Adamas Life, Shanghai, China) was employed to facilitate the extraction of total RNA from human tissue samples. Mechanical disruption of the human tissue was carried out utilizing low-temperature grinders. Subsequent to RNA extraction, the generated RNA was subjected to reverse transcription using a dedicated kit (Yugong Biolabs, Jiangsu, China) to synthesize complementary DNA (cDNA). For the final analysis, the expression levels of the target genes were assessed employing the SYBR Green dye fluorescence quantitative PCR method, a technique known for its sensitivity and accuracy in quantification (Yugong Biolabs, Jiangsu, China). The specific primers applied in this investigation were procured from Sangon Biotech (Shanghai, China). The primers used in this study are as follows: β-actin: (Forward) CCTGGGCATGGAGTCCTGTG; (Reverse) TCTTCATTGTGCTGGGTGCC. PINK1, (Forward) GGAGTATGGAGCAGTCACTTACAG; (Reverse) AGCAGCGGCACGGAAGAG. FAS, (Forward) CAAGTGACTGACATCAACTCCAAGG; (Reverse) GGACAGGGCTTATGGCAGAATTG. CXCR4, (Forward) ACGCCACCAACAGTCAGAGG; (Reverse) AAGTCGGGAATAGTCAGCAGGAG. DAPK1, (Forward) GCTTGGCACGGCTATTACTCTG; (Reverse) CTCTCCTTCTCGGTTCTTGATGTTC; HDAC1, (Forward) GTCGGAGTACAGCAAGCAGATG; (Reverse) CCACAGAACCACCAGTAGACAAC. TP53, (Forward) ATGAGCCGCCTGAGGTTGG; (Reverse) CAGTGTGATGATGGTGAGGATGG. MAPK8IP1, (Forward) AGTGACTCTGCCACCGTCTATG; (Reverse) CCTCCTCATATTCCTCTCCGATGG. CASP8, (Forward) TTTGACCACGACCTTTGAAGAGC; (Reverse) GAGGATACAGCAGATGAAGCAGTC. BNIP3, (Forward) TTCCTTCCATCTCTGCTGCTCTC; (Reserve) AAGGTGCTGGTGGAGGTTGTC. IFNG, (Forward) TTTGGGTTCTCTTGGCTGTTACTG; (Reverse) TTATCCGCTACATCTGAATGACCTG. HSPB8, (Forward) ATGCCCTTCTCCTGCCACTAC; (Reverse) CAAGAGGCTGTCAAGTCGTCTG. ITGB1, (Forward) AGATGTGTCAGACCTGCCTTGG; (Reverse) AATTTGTCCCGACTTTCTACCTTGG.

### Immunohistochemistry of the OARGs

Human Protein Atlas database (HPA, https://www.proteinatlas.org/) aims at creating expressed patterns in protein of cells as well as tissues [32]. We can download immunohistochemistry images of GC and normal tissues via HPA platform.

### Cluster analysis of OARG

Consensus cluster was carried out utilizing “ConsensusClusterPlus” R package for GC samples based on the expression of 17 OARGs to detect distinctive OARG modification patterns [33]. The ideal clustering number was established based on the cumulative distribution function (CDF) curve as well as variations of CDF curve area. Principal component analysis (PCA) was used to demonstrate the accuracy of our clustering findings. Then the prognosis and clinical pathological features between different subtypes were further compared.

### Gene set variation analysis (GSVA) and single sample gene set enrichment analysis (ssGSEA)

The GSVA was carried out using the “GSVA” R tool in order to discover biological process diversity across different OARG modification patterns [34]. We then evaluated the variations in infiltrated levels of immune cell subset between different subtypes. The relative abundance of each immune cell in each sample was represented by the enrichment scores obtained from the ssGSEA analysis [35, 36].

### Differentially expressed genes (DEGs) were analyzed to determine the patterns of OARG modification

According to three different OARG modification patterns that we have concluded, we screened out DEGs among GC patients by the R package “limma” with adjusted *P* < 0.001 as the criterion [37]. In order to unravel the biological processes and functions DEGs are involved in, the “org.Hs.eg.db”, “clusterProfiler”, “enrichplot”, and “ggplot2” packages were utilized to perform GO and KEGG enrichment analyses [38]. The univariate Cox analysis was used to identify prognostic DEGs, and the R program “ConsensusClusterPlus” was used to conduct a cluster analysis based on the prognostic DEGs [39]. Additionally, changes in OARG expression and the survival of various genotypes were examined.

### Construction of OARG gene signature

Following that, we developed an OARG scoring system based on the results. The PCA of principle components was performed, and we selected principal components 1 and 2 as feature scores. It is largely concentrated in the score of the gene block with the most significant correlation or inverse correlation. In the meanwhile, it took into account the extent the impact of untracked genes on other set members. The following equation was used to calculate the OARGscore: OARGscore = Ʃ(PC1i+PC2i). i in the equation stands for genes connected to the OARG phenotype [40].

### Genomic information of immune checkpoint genes (ICGs)

We compared the differential expression of ICGs like CTLA4, LAG3, CD40, CD80, CD86, and CD276 in groups with low- and high-OARGscore. We also acquired the Immune Checkpoint Inhibitor (ICI) Immunophenoscore (IPS) dataset from The Cancer Immunome Atlas Database [35]. The immunotherapeutic implications of the OARGscore were investigated using IPS, a reliable tool for assessing tumor immunogenicity [41].

### OARGscore and chemotherapeutic drug correlation

Data regarding the therapeutic sensitivity of cancer cells and molecular markers of drug response can be found in a public dataset called Genomics of Drug Sensitivity in Cancer (GDSC) [42]. An R tool called “OncoPredict” predicts drug response *in vivo* or in cancer patients from GDSC [43].

### Statistical analysis

All data analysis and visualization were done with R software. The best cutoff score was divided into groups with low- and high-OARGscore using the “surv-cutpoint” function. The Kaplan-Meier (KM) method was used to create survival curves for progression experiments. The TCGA-STAD cohort’s patients in the high- and low-OARGscore groups were presented with their mutations using the waterfall function of the maftools package. *P* < 0.05 was regarded as statistically significant in all bilateral statistical *p* values.

### Availability of data and materials

The datasets analyzed in this work may be found in the TCGA and GEO databases.

## RESULTS

### Data procession

**Figure 1:**
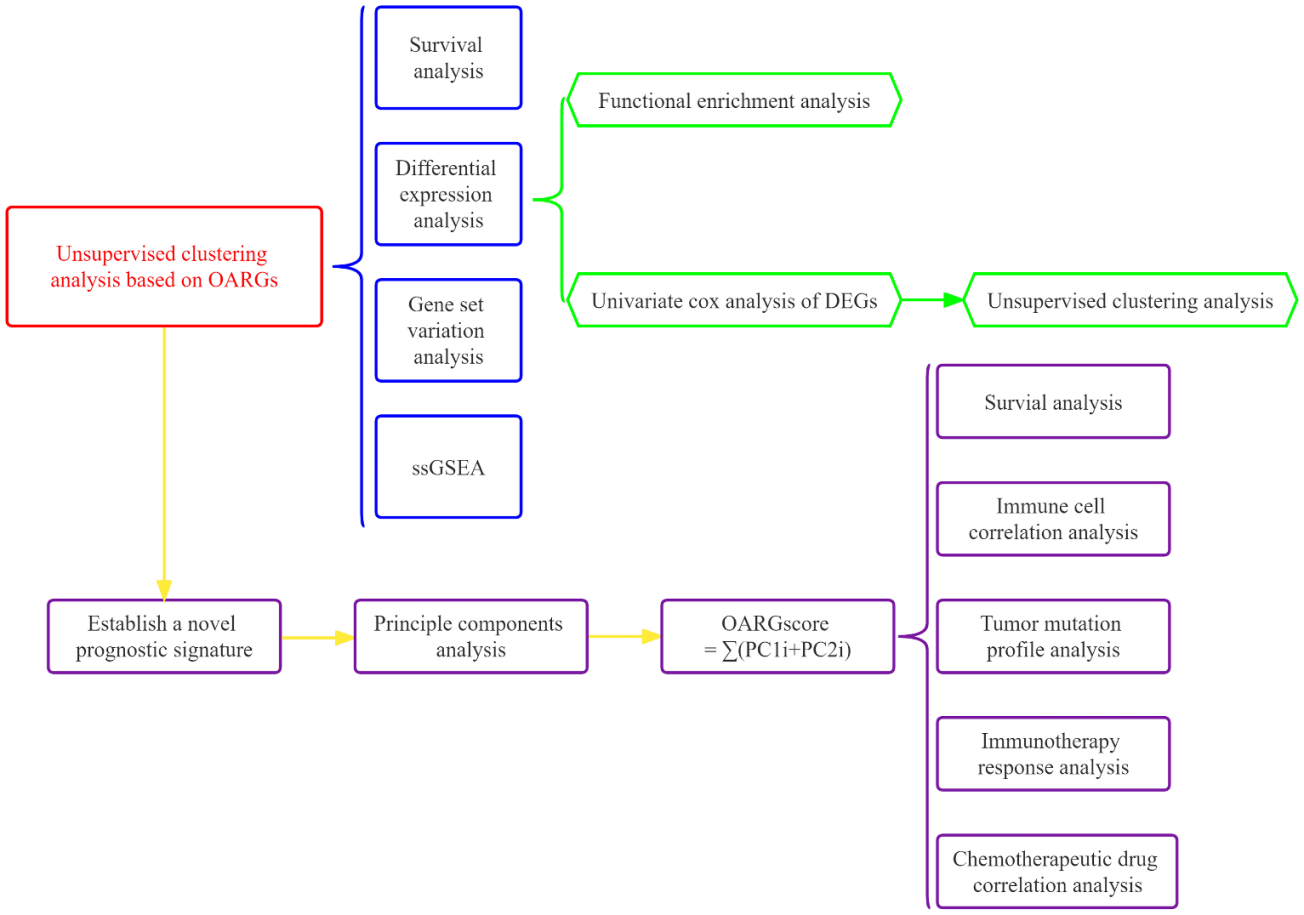
The investigation’s flow chart.

Figure 1 displayed the flowchart for the research. In order to get 96 OARGs, we first took the intersection of genes associated to OS and genes connected to autophagy (Supplementary Figure 1). A univariate Cox regression analysis was then performed on the 808 GC samples to produce 17 prognostically significant OARGs for this study’s analysis.

### Landscape of the genetic variation of OARG in GC

**Figure 2:**
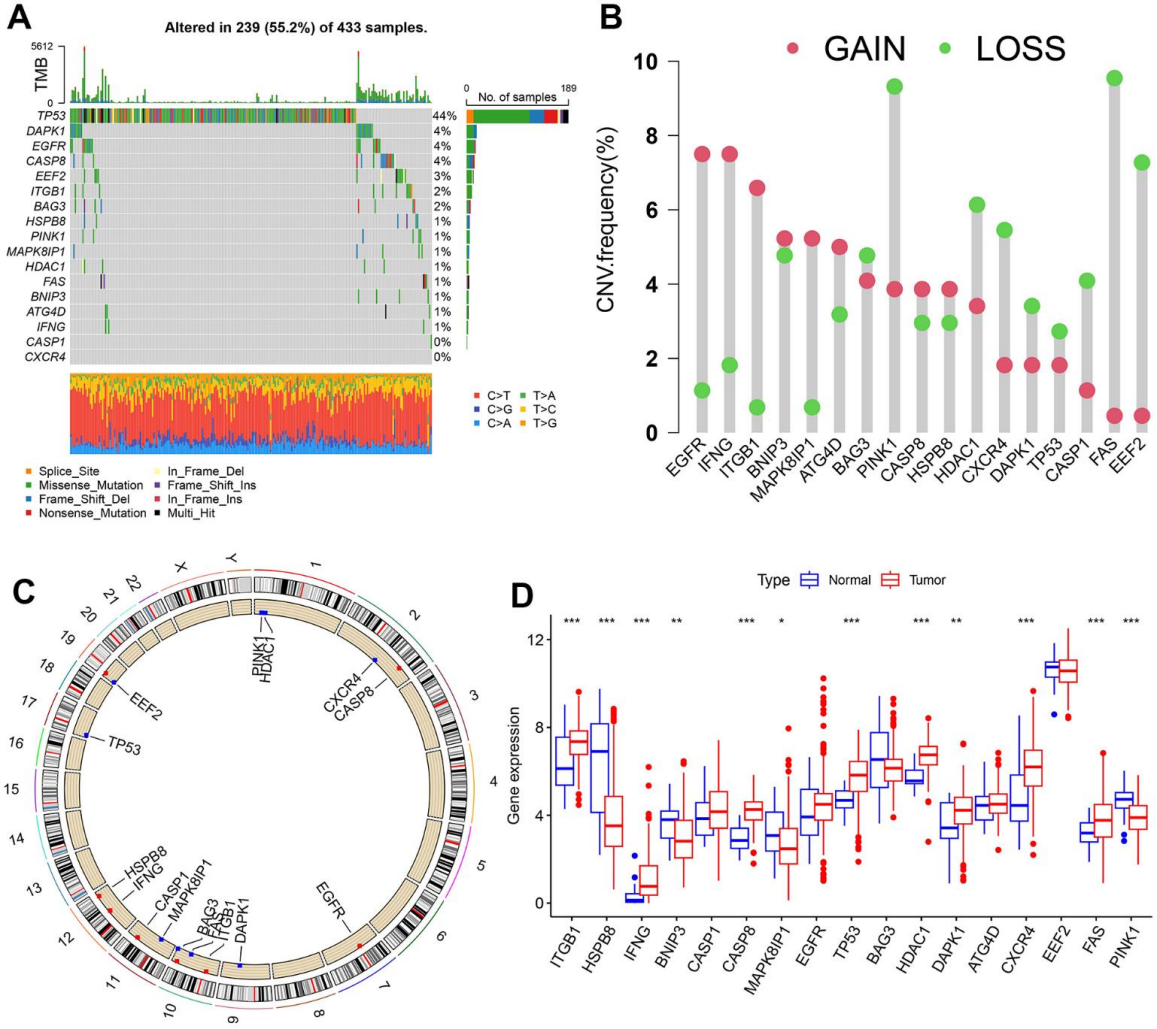
**Landscape of genetic and expression variation of OARGs in the TCGA-SATD cohort.** (**A**) The frequency of mutation for 17 OARGs. (**B**) CNV variation frequencies of OARGs. (**C**) Chromosomal locations of altered CNV in the OARGs. (**D**) The difference in 17 OARGs expression between healthy tissue and malignant tissue.

17 OARGs were ultimately found in this study and included for subsequent studies. The frequency of copy number variations (CNVs) and somatic mutations in the GC samples were compiled to obtain a thorough overview of the genetic variation of OARGs in GC. 239 (55.2%) of the 433 samples had OARG mutations, with TP53 having the highest mutation frequency followed by DAPK1, EGFR, and CASP8. However, CASP1 and CXCR4 did not have any mutations (Figure 2A). The analysis of CNV alteration frequency revealed extensive CNV alteration in 17 OARGs, the majority of which were centered on copy number deletion, while CNV amplification was frequently observed in EGFR, IFNG, ITGB1, BNIP3, MAPK8IP1, ATG4D, CASP8, and HSPB8 (Figure 2B). The location of CNV alteration of OARGs on chromosomes was shown in Figure 2C. Additionally, we evaluated the mRNA expression levels of 17 OARGs across cancer and normal samples in order to determine the relationships between genetic variants and the expression of OARGs. The findings revealed that whereas HSPB8, BNIP3, MAPK8IP1, and PINK1 expression levels were decreased in tumor tissues, those for ITGB1, IFNG, CASP8, TP53, HDAC1, DAPK1, CXCR4 and FAS were up (Figure 2D). Following these, the expression profiles of 12 differentially expressed OARGs were validated by RT-PCR and immunohistochemistry in clinical samples of GC (Supplementary Figure 2A, 2B). Due to the lack of protein expression information for CXCR4 in the HPA database, only the other 11 available OARG proteins were examined. These results were generally consistent with the bioinformatics results described above, and some of the differences may be due to the small sample size and the heterogeneity among tumors.

### Three OARG patterns of GC

**Figure 3:**
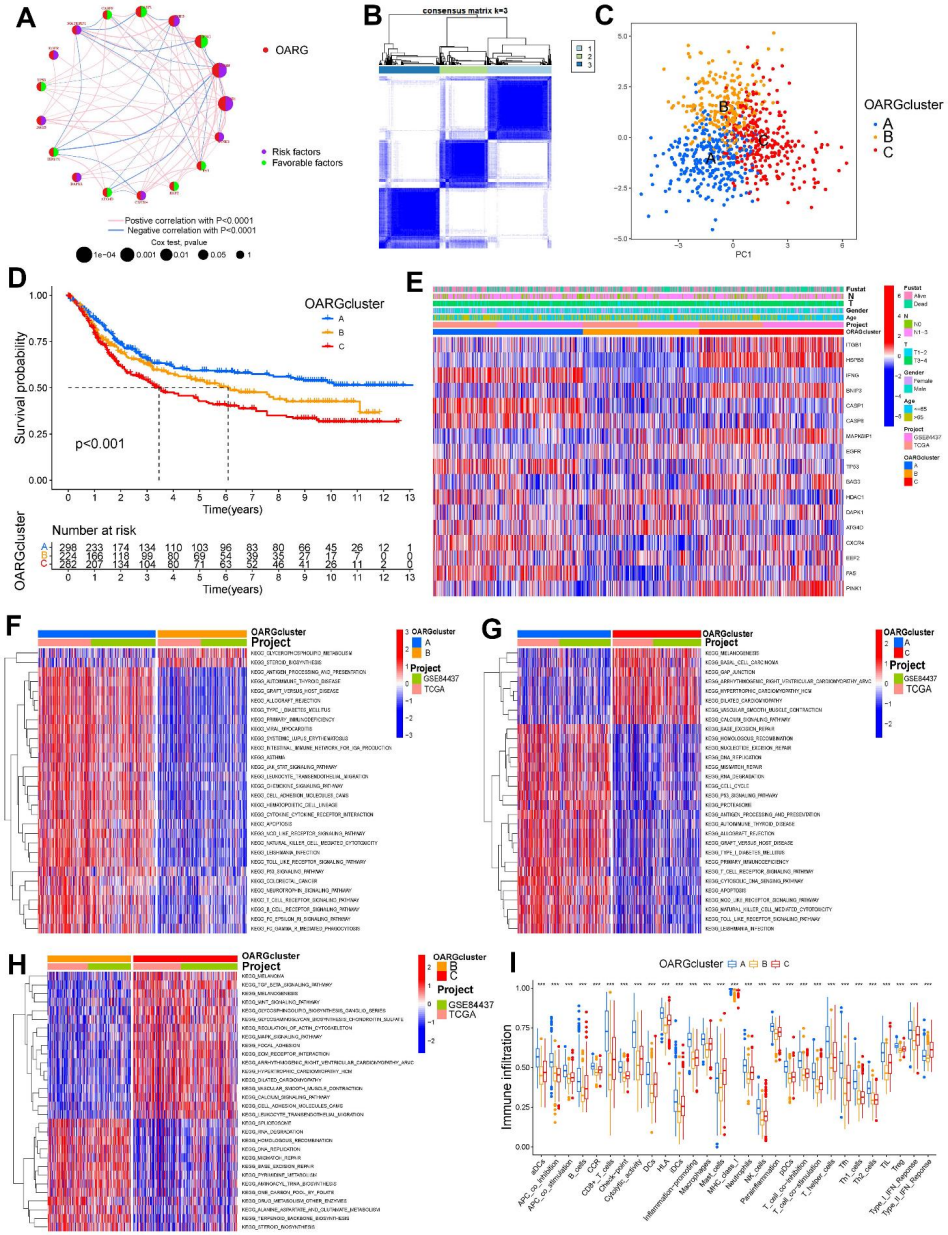
**OARG modification patterns and the biological characteristics of each pattern.** (**A**) The interaction of OARGs. (**B**) Consensus matrix. (**C**) Principal component analysis for three OARG patterns’ transcriptome profiles. (**D**) Survival analyses for the three OARG patterns. (**E**) Clinical characteristics of three OARG modification patterns. (**F**–**H**) The activation statuses of biological pathways are displayed in different OARG modification patterns. (**I**) The infiltration levels of immune cell subsets in three OARG modification patterns.

We integrated the expression data of GC samples using correlation and univariate regression analysis to investigate the association between the expression of OARGs and the prognosis of GC (Figure 3A). Then, depending on the expression of OARGs, GC patients were grouped using consensus clustering. GC patients could clearly be identified when cluster number was three (Figure 3B, 3C and Supplementary Figure 3). Cluster A greatly outperformed Cluster B and Cluster C in terms of prognosis among the three molecular subtypes that were identified (Figure 3D). Figure 3E illustrated the clinical characteristics of the three clusters. Also, we evaluated at the enriched pathways in the three subtypes using GSVA. In KEGG pathways, cluster A significantly upregulated a number of immunity-related pathways, including T cell receptor signaling, FC gamma mediated phagocytosis, chemokine signaling, p53 signaling, and Toll like receptor signaling (Figure 3F–3H). The pathways for MAPK signaling, TGF BETA signaling, leukocyte transendothelial migration, and cell adhesion molecules were particularly enriched in Cluster C (Figure 3F–3H). Additionally, we examined the immune cell infiltration of the various clusters and discovered that, in addition to mast cells and type II IFN responses, cluster A had a high level of infiltration of the remaining immune cells (Figure 3I).

### DEGs between distinct OARG phenotypes

**Figure 4:**
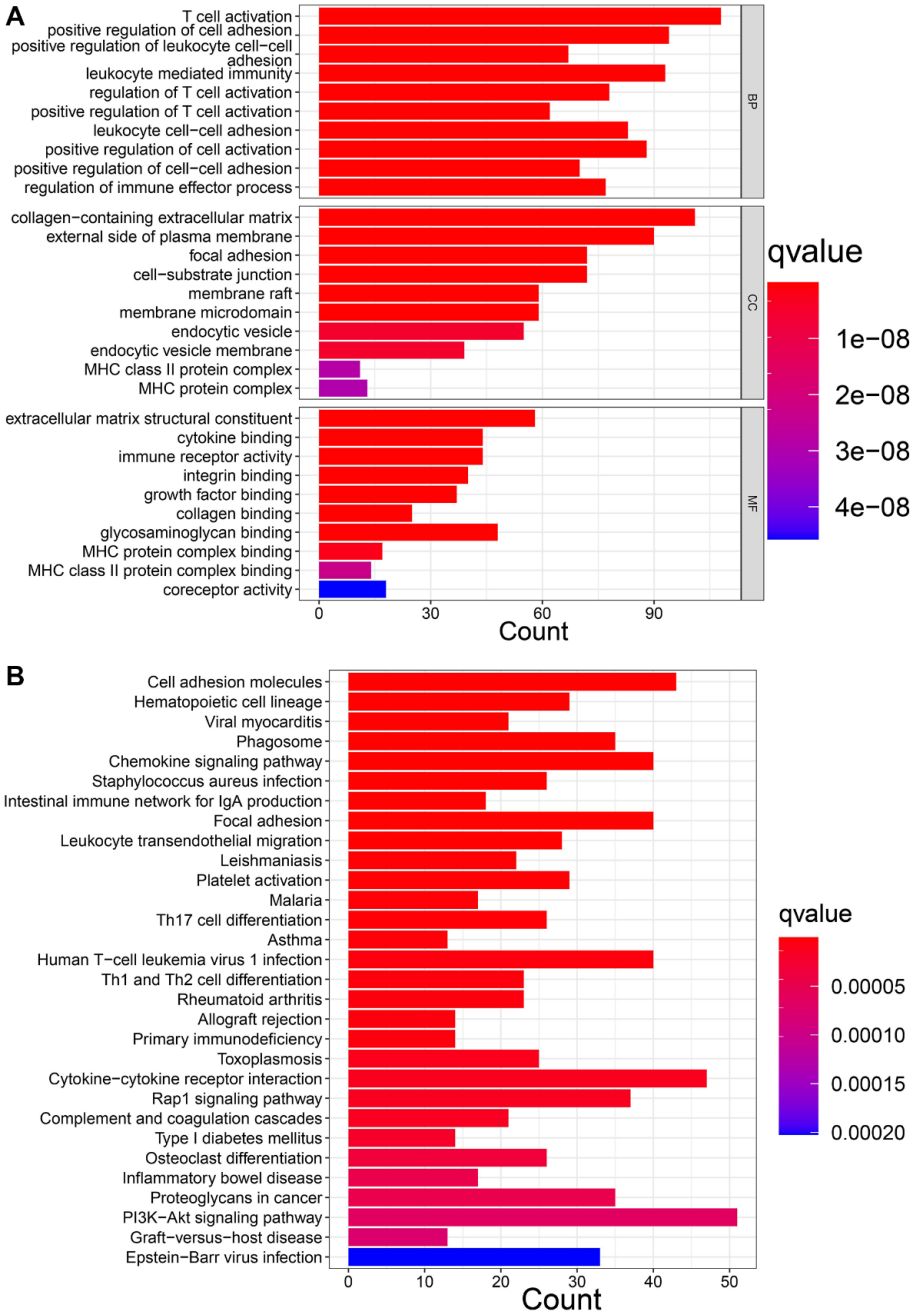
**Function enrichment analysis of DEGs.** (**A**) GO enrichment analysis. (**B**) KEGG enrichment analysis.

**Figure 5:**
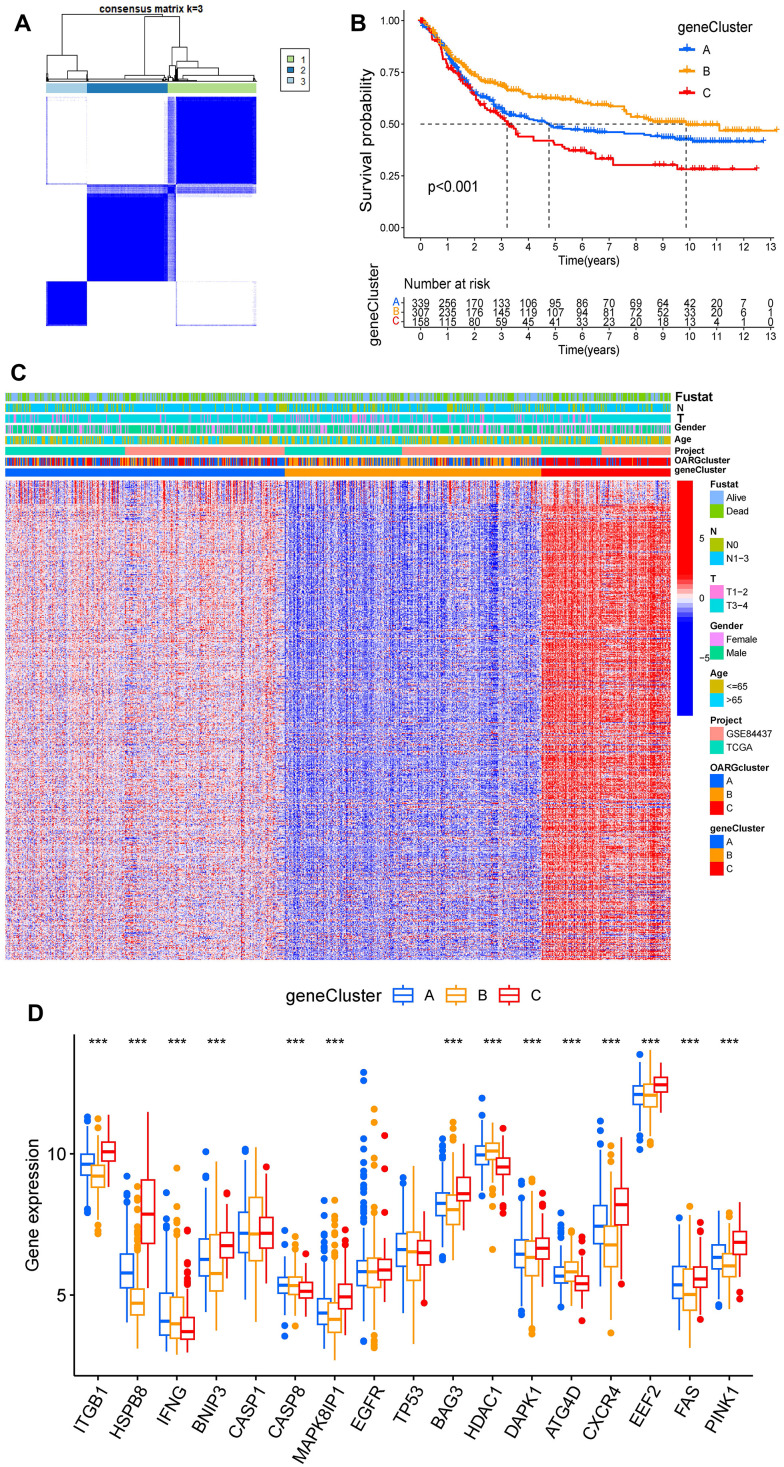
**Identification of subtypes based on DEGs.** (**A**) Consensus matrix heatmap when cluster number k = 3. (**B**) KM analysis of GC patients in three geneClusters. (**C**) These subgroups’ various clinicopathological traits are displayed by a heatmap. (**D**) The expression of 17 OARGs in three geneClusters.

We then screened DEGs between the three subtypes since clusters A, B, and C showed notable variations in overall survival, tumor microenvironment, and enriched pathways. The “limma” program was used to filter 1273 DEGs. Figure 4A, 4B display the findings of the GO and KEGG pathway enrichment analyses of DEG. These genes are primarily concentrated in pathways that connect to tumors and immunological function, such as immune receptor activity, T cell activation, leukocyte-mediated immunity, and regulation of T cell activation. The DEGs’ prognostic significance was examined using a single-variable Cox regression analysis, and 613 genes were discovered to be associated with overall survival. Based on 613 prognostic DEGs, patients were again divided into three subgroups (geneClusters A, B and C) using unsupervised clustering to further explore the different OS and autophagy-related modification patterns in GC (Figure 5A and Supplementary Figure 4). Patients with GC in geneCluster B fared better than those in geneCluster A and C in terms of survival (Figure 5B). Figure 5C displays the various clinicopathological characteristics of these groupings. Additionally, Figure 5D demonstrated the expression levels of 17 OARGs in different geneClusters.

### Construction of OARGscore

**Figure 6:**
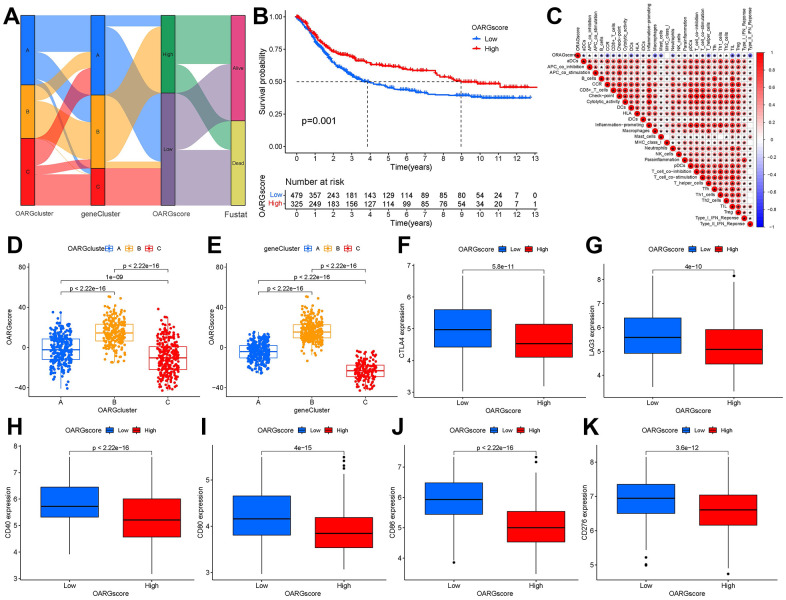
**Creation of OARG signature and study of its clinical implications.** (**A**) The Sankey diagram showed the relationship between the survival status of GC patients and the OARGcluster, geneCluster, and OARGscore. (**B**) Survival outcomes of patients by OARGscore. (**C**) Association between immune cells and OARGscore. (**D**) Analysis of OARGscore variation between OARGclusters. (**E**) Analysis of OARGscore variation among gene clusters. (**F**–**K**) Expression of immune checkpoints (CTLA4, LAG3, CD40, CD80, CD86 and CD276) between low- and high-OARGscore groups.

Based on these phenotype-related genes and taking into account the individual variety and complexity of OARG modification, we developed a set of scoring systems that we called OARGscore to measure the OARG modification pattern of specific GC patients. The attribute changes of specific patients were represented by an alluvial diagram (Figure 6A). We then examined prediction of patient survival outcomes based on OARGscore. High-OARGscore patients have higher survival rates (Figure 6B). We also looked at the relationship between the TME and OARGscore. Higher OARGscores showed lower immune cell infiltration levels because the OARGscore was inversely connected with the majority of immune cells (Figure 6C). We chose a few significant ICGs, such as CTLA4, LAG3, CD40, CD80, CD86, and CD276 and assessed the expression of each in groups with high- and low-OARGscore. According to the results (Figure 6F–6K), the low-OARGscore group had higher expression levels of all six ICGs, indicating that suggesting that these ICGs may be potential therapeutic targets. The OARGscore was then used to perform survival analyses, categorize patients by sex, age >= 65 years, T stage, and N stage. It shows that our model is meaningful in different clinical features, according to the findings (Supplementary Figure 5).

### Patterns of OARG modification in TCGA molecular subgroups and cancer somatic mutations

**Figure 7:**
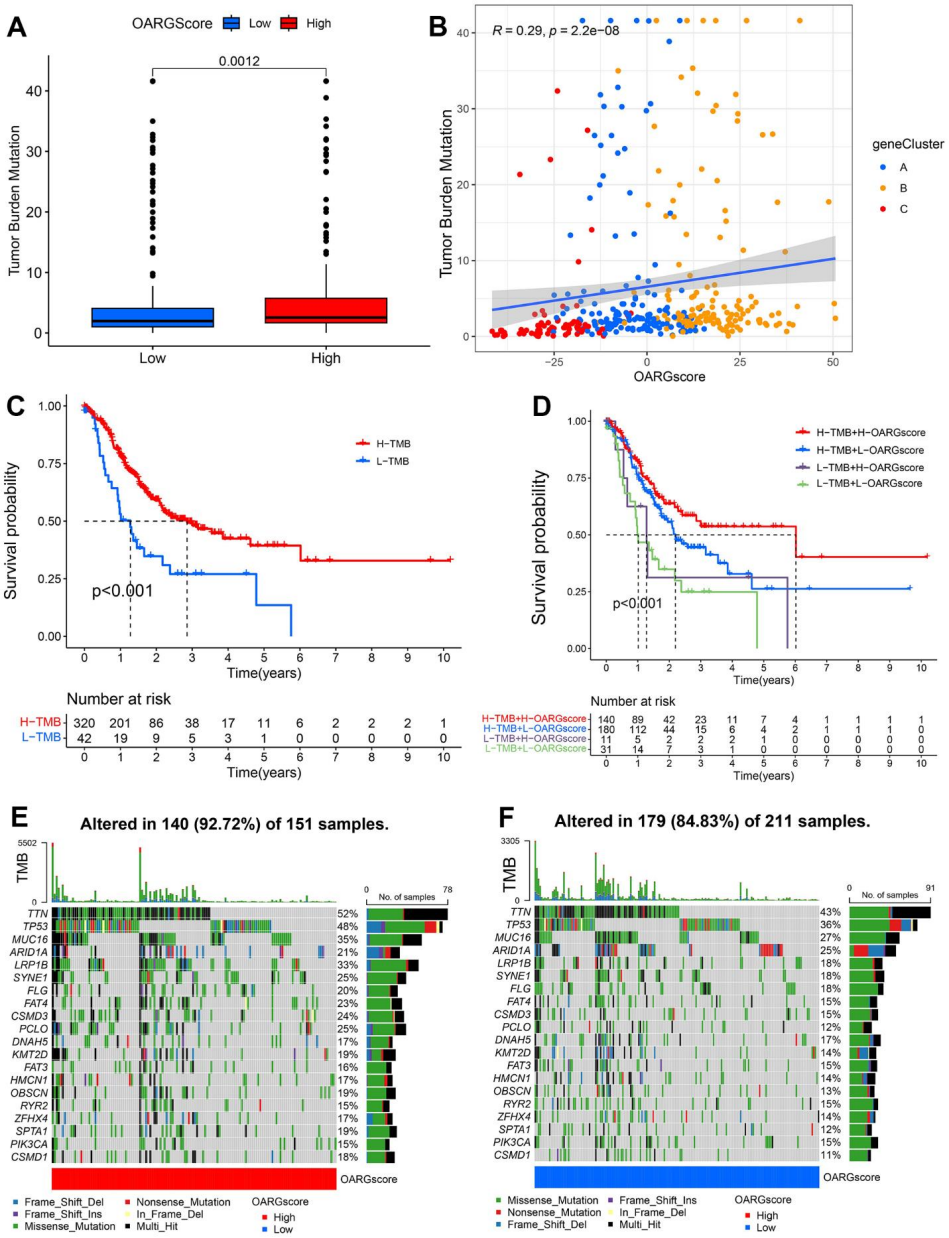
**Characteristics of OARG modification in cancer somatic mutation.** (**A**) The differences in the TMB between low- and high-OARGscore groups. (**B**) The relationship between the TMB and OARGscore. (**C**) Survival analysis utilizing KM curves for low- and high-TMB groups. (**D**) KM curves for patients stratified by both TMB and OARGscore. (**E**, **F**) Waterfall plot of cancer somatic mutations constructed from patients with (**E**) high-OARGscore and (**F**) low-OARGscore.

There is growing evidence that links somatic mutations in tumor genomes to the response to immunotherapy. The distributions of tumor mutation burden (TMB) among the various OARGscore groups were therefore examined. Figure 7A shows that the high-OARGscore group had a greater TMB than the low-OARGscore group and that the OARGscore was positively correlated with TMB (Figure 7B). Additionally, patients with large mutational burdens had a noticeably better likelihood of surviving (Figure 7C). Likewise, we discovered that high-OARGscore group with a high TMB demonstrated greater survival (Figure 7D). Then, we assessed the distribution of common gene mutations in the high- and low-OARGscore populations. Twenty genes with the highest mutation frequency in two groups were shown in Figure 7E, 7F. In conclusion, somatic mutations and OARG changes interact, and the categorization of OARGscore may be influenced by the variation. These results indicated that patients with high-OARGscore group could benefit more from immunotherapy.

### OARGscore and immunotherapy

**Figure 8:**
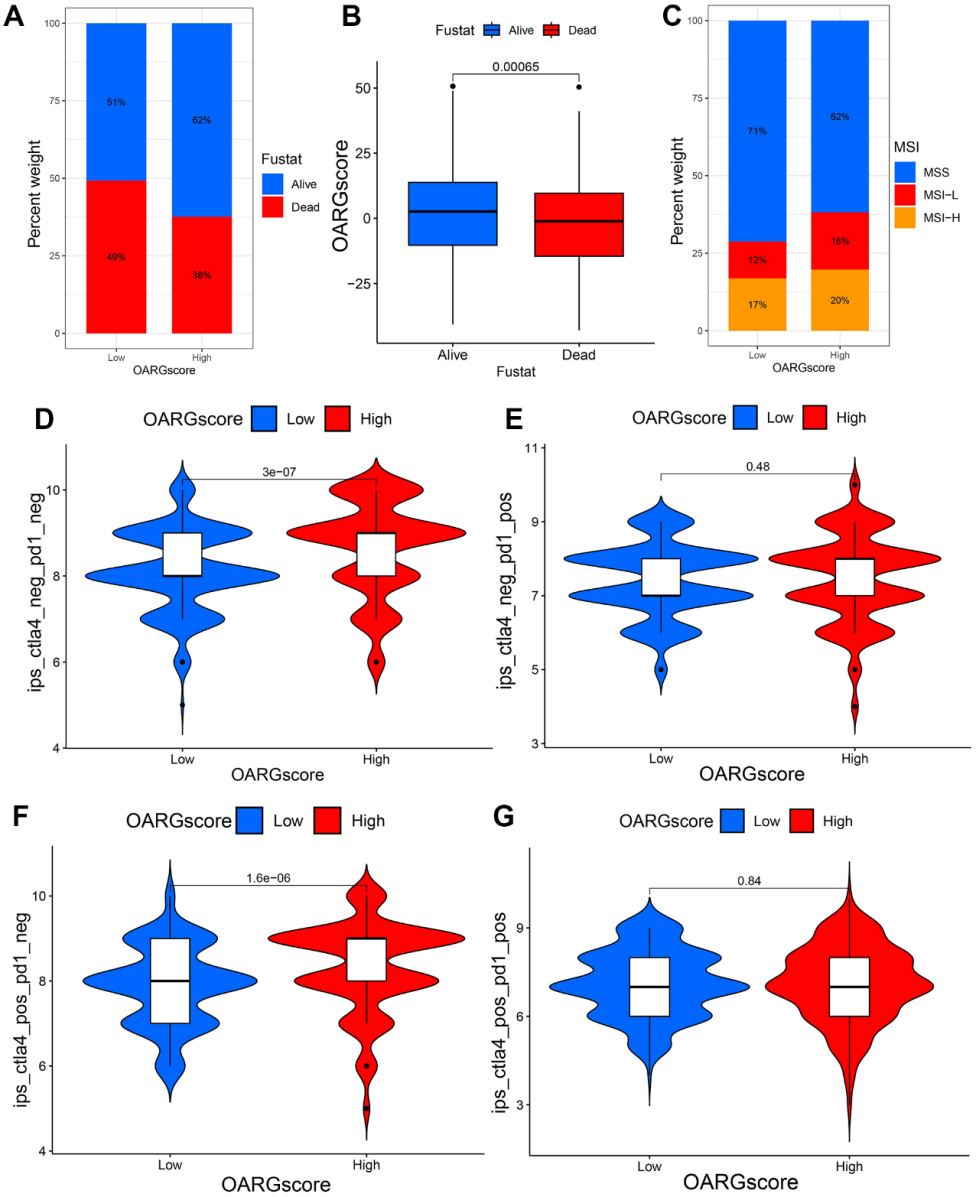
**OARGscore in the role of immunotherapy.** (**A**, **B**) The percentage of patients who survived in groups with low- and high-OARGscore. (**C**) The proportion of MSI grouping in low and high-OARGscore groups. (**D**–**G**) A comparison of the IPS relative distribution across groups with low- and high-OARGscore.

We looked at the effect of OARGscore in predicting immunotherapy response in the TCGA cohort since patients had comprehensive immunotherapy information. The findings indicate that individuals with high OARGscore experienced considerable therapeutic advantages and had significantly higher survival rate (Figure 8A, 8B). Microsatellite instability-high (MSI-H) is a potential predictor of immunotherapy response targeting PD-1 or its ligand PD-L1 [44]. We found that MSI-H made up a sizable amount of the high-OARGscore group (Figure 8C). The effects of CTLA-4/PD-1 inhibitor therapy were different for the groups with high and low-OARGscore, as shown in Figure 8D–8G. The high-OARGscore group had higher IPS scores, indicating that they were predicted to be more immunogenic on ICIs and more likely to benefit from immunotherapy. These findings imply that OARGscore can be employed to forecast patient immunotherapy response.

### OARGscore guided chemotherapy strategies

**Figure 9:**
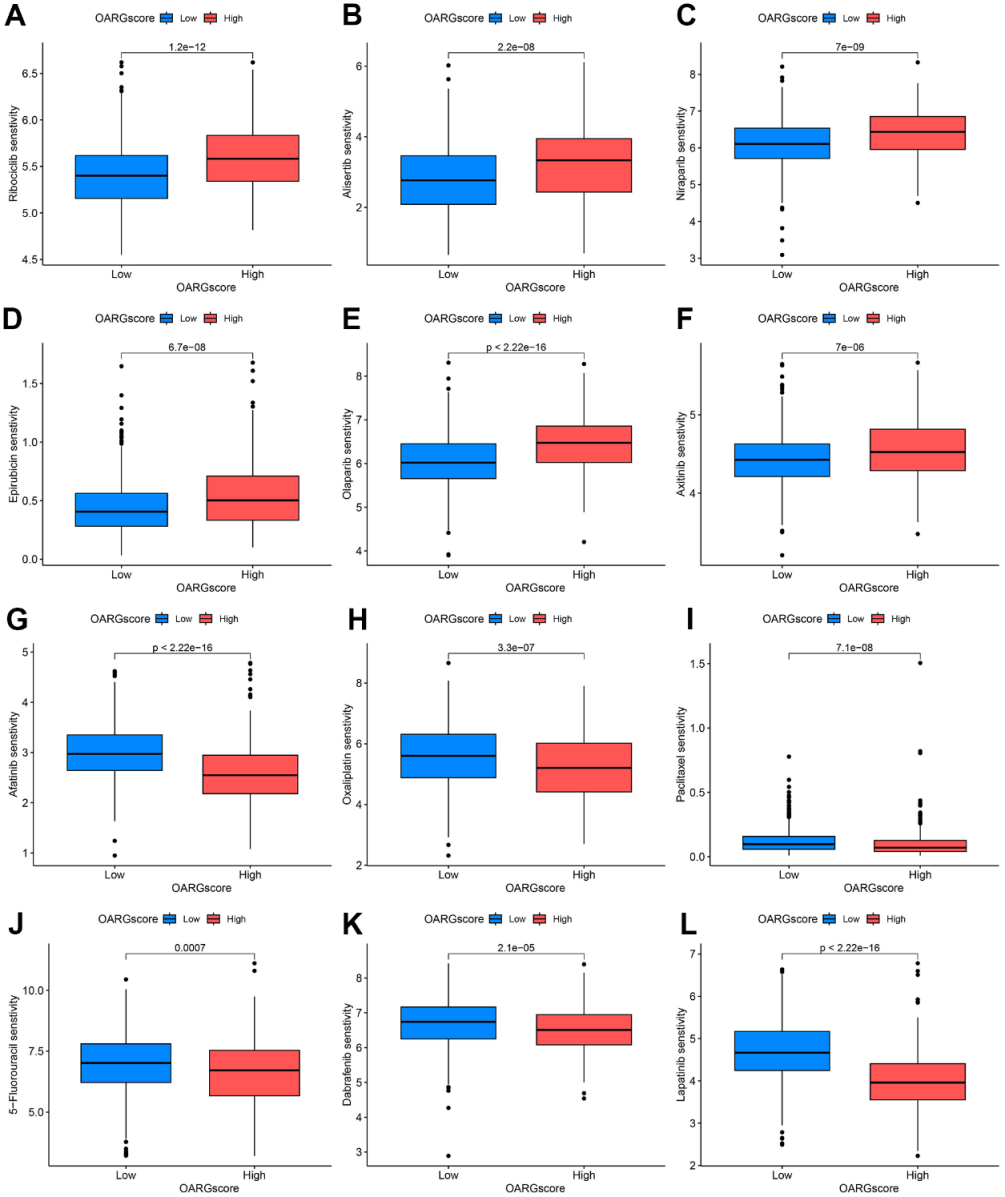
**OARGscore guided chemotherapy strategies.** (**A**–**F**) Predicted sensitivity of Ribociclib, Alisertib, Niraparib, Epirubicin, Olaparib, and Axitinib, which were candidate chemotherapeutic agents for low-OARGscore patients. (**G**–**L**) Predicted sensitivity of Afatinib, Oxaliplatin, Paclitaxel, 5−Fluorouracil, Dabrafenib and Lapatinib, which were candidate potent drug options for high-OARGscore patients.

Given that chemotherapy is also an effective method for the treatment of GC, it has important clinical application value and prospects. We therefore investigated whether the OARGscore could predict drug sensitivity in GC patients. We found that low-OARGscore group was predicted to benefit more from Ribociclib, Alisertib, Niraparib, Epirubicin, Olaparib, and Axitinib (Figure 9A–9F), while patients in the high-OARGscore group was predicted to benefit more from Afatinib, Oxaliplatin, Paclitaxel, 5−Fluorouracil, Dabrafenib and Lapatinib (Figure 9G–9L).

## DISCUSSION

China accounts for about 50% of GC cases, and the majority of these cases are discovered at a late stage [45]. Although advancements in the use of multimodal of treatment for GC, the total survival rate in the patient population is still far from ideal [46]. The best ways to reduce GC death rates at the moment are suggested to be prevention and individualized treatment [47]. Therefore, a possible progress in improving the prognosis and extending the survival in people of GC could result from the development of new and practicable prognostic biomarkers and treatment targets.

In our bodies, oxidation takes place constantly. The main products of OS are ROS, also known as free radicals. They harm molecules that make up DNA, proteins, and lipids in order to acquire higher stability, which causes tissue damage [25]. There is a lot of proof that the body's ongoing production of ROS can both encourage and prevent cancer cells from surviving [48]. Numerous tumors have been found to produce increased ROS, and it has been demonstrated that this has a range of impacts. For instance, they could increase cell survival and proliferation, activate protumorigenic signals, and facilitate genetic instability and DNA damage, among other things. What’s more, ROS can encourage antitumor transmission of signals and start OS-induced tumor death of cells. ROS disturb the redox equilibrium between cancerous cells and normal ones, which raises the possibility that ROS could be a target for tumor immunotherapy. Autophagy is a multistep lysosomal breakdown system that is highly regulated. To encourage the metabolism and renewing for the cells, it breaks down injured organelles, unfolded proteins, and toxic substances and delivers those to the lysosome for digesting [49]. Autophagy can play a dual part in carcinoma. On one hand, as a divisor of tumor suppression, it limits the buildup of damaged organelles and proteins. On the other hand, it is also a cell’s surviving mechanism, promoting the growth of established tumors [50]. It has also been found that autophagy is closely related to anti-cancer immunity [51, 52]. A large body of evidence suggests that OS is closely related to autophagy, and that ROS and RNS are the main intracellular signaling sensors that maintain autophagy [25]. Therefore, it is promising to research the molecular mechanism and immunotherapy of OARGs related to GC.

A total of 17 OARGs that associated with the prognosis of GC were included in our analysis. Firstly, somatic mutations, copy number changes and expression levels of OARGs in GC patients were preliminarily evaluated, and it was found that most OARGs had copy number changes and differential expression. These findings suggest that the imbalance of OARG expression was highly correlated to GC incidence and development. Concentrating on the way distinct OARGs interact with one another, cross-talk in OARGs might be important in the formation of different OARG modification patterns and TME cell-infiltrating characteristics in specific malignancies.

Besides that, GC samples were clustered into three subgroups with various biological behaviors and TME features based on the expression of OARGs. Cluster A has the best prognosis and is enriched in immune-related pathways. Higher immune infiltration might be the potential cause for more favorable prognosis. To further explore the modification modes of different OARGs in GC, we conducted differential analysis on three clusters, and functional enrichment analysis indicated that these genes were mainly linked to immune-related functions as well as some cancer- associated pathways. Then we carried out consensus cluster analysis again based on the prognosis-related DEGs. Interestingly, GC patients were again divided into three categories with significant prognostic differences. These results indicate that there may indeed be three different OARG-related modification modes in GC. GC patients with different OARG-related modification modes had distinctly varied clinical and transcriptome features.

However, besides to the aforementioned evaluations of the patient population, a scoring system called OARGscore that quantifies the OARG modification pattern was developed based on these OARG signature genes because of the unique variety and complexity of the OARG modification in different patients. The link between OARGscore and immune cell infiltration were analyzed to further investigate a possible relationship between OARGscore and TME. The result indicated that OARGscore was inversely linked to the degree of immune cell infiltration. High OARGscore were found to be associated with a higher rate of survival in patients. ICG expression was elevated in the group with a low-OARGscore, meanwhile. As everyone known that tumor cells may defend themselves through the immunological checkpoint pathway and are incorrectly believed to be a normal component of the body. The low-OARGscore group with a larger percentage of immunological components had a poorer prognosis, demonstrating the activation of ICG mechanisms. A possible GC target might be the over expression of CTLA4, LAG3, CD40, CD80, CD86, and CD276.

The efficacy of immunotherapy in GC has been variable because of a lack of a thorough knowledge of the immunological environment in GC and the difficulty to pinpoint a specific patient’s immune status. According to reports, TMB can be utilized as a predictor of immunotherapy effectiveness and has evolved into a biomarker among certain kinds of cancer for identifying people who can profit from immune therapy [53, 54]. This might be because there’s a chance that elevated TMB levels will result in more neoantigens that the immune system will detect and start a stronger anti-tumor immunological reaction [55]. Microsatellites are short tandem repeats with a high mutation rate that are dispersed across the entire genome and range in length from 1-6 nucleotides. MSI is therefore defined as a hyper-mutable circumstance that develops at genomic Microsatellite in the presence of a deficient DNA mismatch repair machinery [56]. MSI-H cancers are linked to elevated tumor-infiltrating lymphocytes and enriched PD-L1 expression across tumor types. Our research revealed that TMB is higher in the high-OARGscore group, and a survival analysis revealed that people who have high TMB expression among the same OARGscore group had a higher survival rate. Moreover, the proportion of MSI-H in high-OARGscore was larger than that in low-OARGscore. This finding demonstrates that the high-OARGscore subgroup of patients may gain more from immunotherapy. After that, more research was done to determine the relative IPS distribution between the groups with low- and high-OARGscore. Again, the results show that high scores were predicted to benefit from immunotherapy.

Additionally, the OARGscore can forecast the susceptibility to chemotherapeutic drugs. Low-OARGscore group was predicted to benefit more from Ribociclib, Alisertib, Niraparib, Epirubicin, Olaparib, and Axitinib, while patients with high-OARGscore was predicted to benefit more from Afatinib, Oxaliplatin, Paclitaxel, 5−Fluorouracil, Dabrafenib, and Lapatinib.

To our knowledge, this is the first instance in which the role of OS and autophagy in GC prognosis, immunotherapy, and chemotherapy has been examined using bioinformatics. However, there are certain limitations to this analysis. First off, further prospective studies need be conducted to corroborate these findings since the research was retrospective analysis. Second, there is no *in vitro* or *in vivo* testing to validate the veracity for the mechanistic analyses in the evidence. As a result, several research will be carried out in the future to show the molecular relationships among these genes and GC development.

## CONCLUSIONS

This study showed that OARG alteration patterns are crucial in determining the complexity and diversity of TME. The OARGscore was established to identify the OARG modification mode of individual patients, which can effectively predict the prognosis, TME, immunotherapy response and chemotherapy drug sensitivity for people of GC.

## Supplementary Material

Supplementary Figures
