# Supplementary material for: Oxidative stress and autophagy-mediated immune patterns and tumor microenvironment infiltration characterization in gastric cancer
Source: Aging (Albany NY). 2023 Nov 9;15(21):12513–36. doi: 10.18632/aging.205194 (PMC10683600; doi:10.18632/aging.205194)
Supplement: Supplementary Figures [file aging-15-205194-s001.pdf]

SUPPLEMENTARY FIGURES

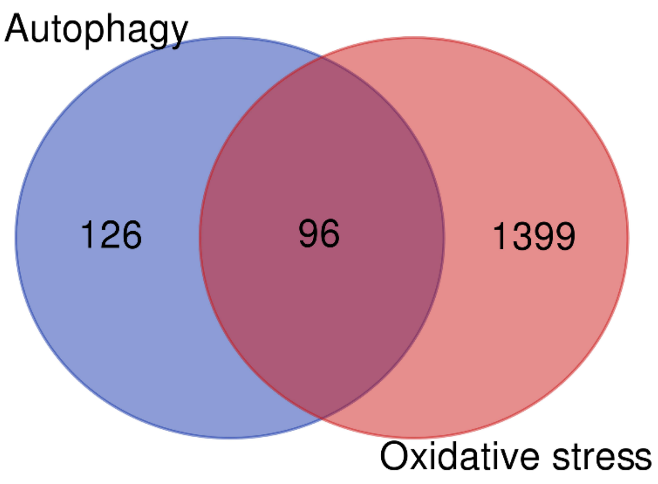

Supplementary Figure 1. Venn diagram to find 96 OARGs.

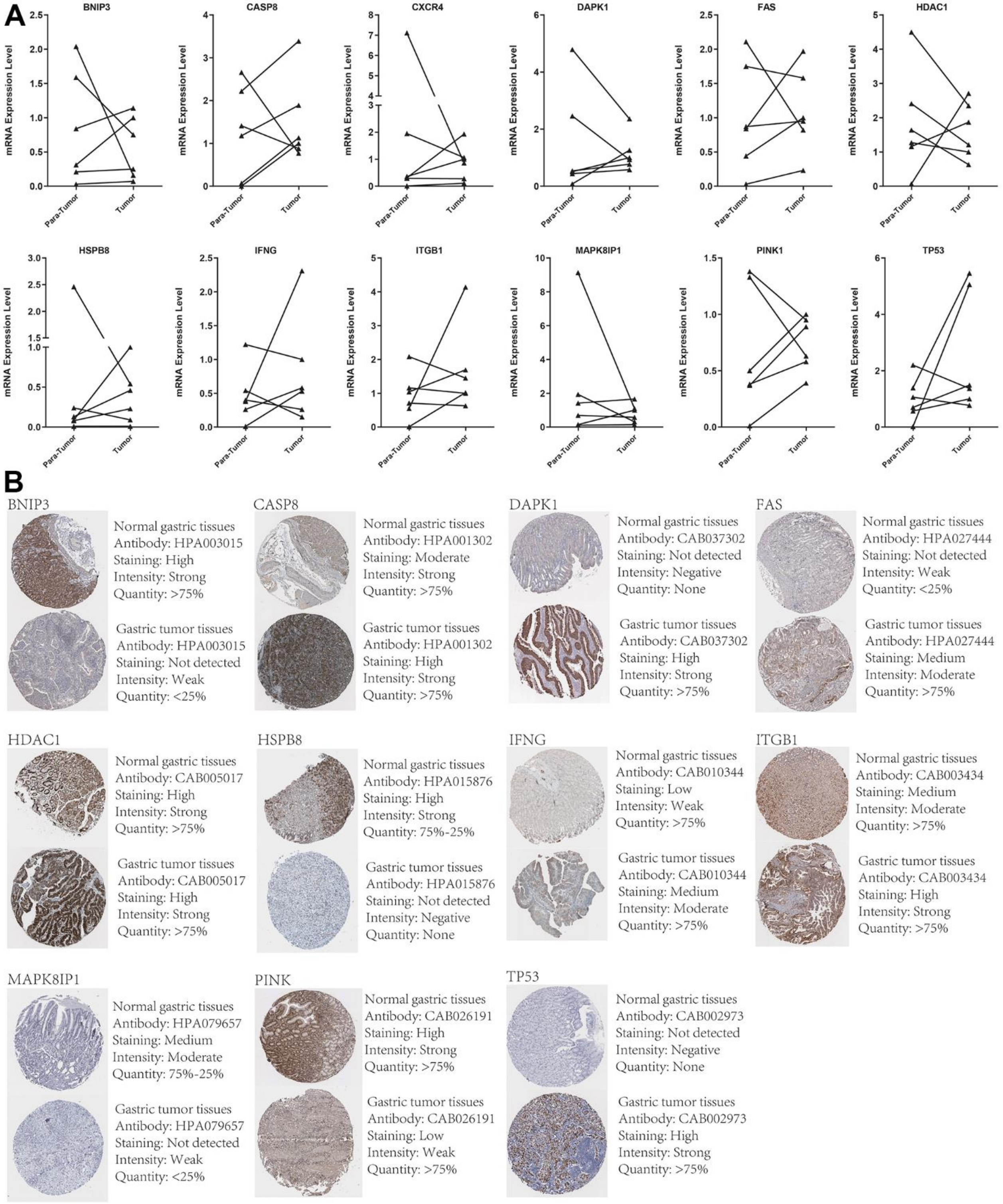

Supplementary Figure 2. Validation of the expression traits of OARGs. (**A**) RT-PCR of clinical samples (tumor tissues vs normal adjacent tissue). (**B**) Immunohistochemistry of clinical samples.

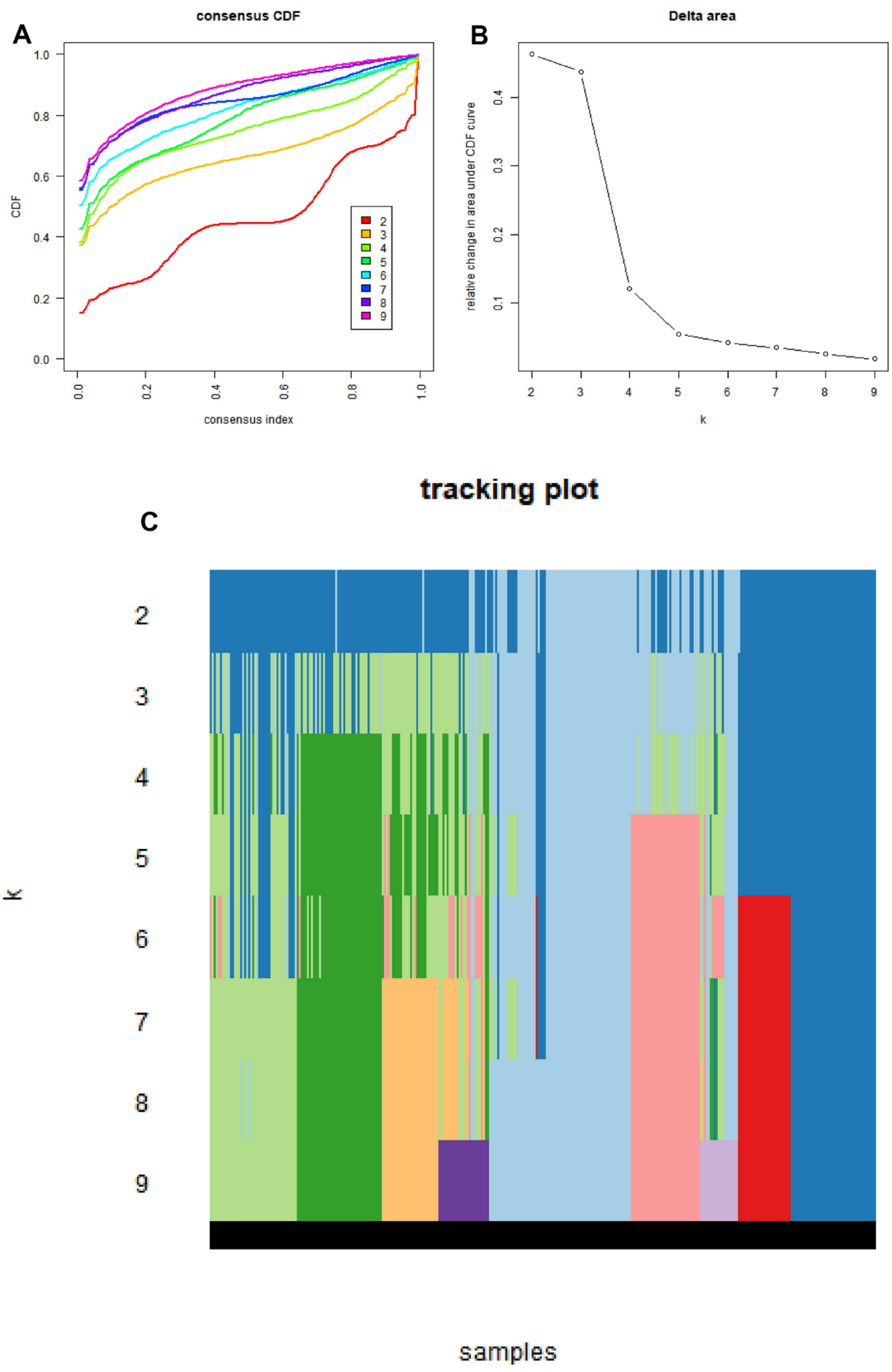

Supplementary Figure 3. Unsupervised cluster analysis to establish OARG clusters. (**A**) CDF cumulative distribution curve. (**B**) Area under the CDF curve. (**C**) Tracking plot.

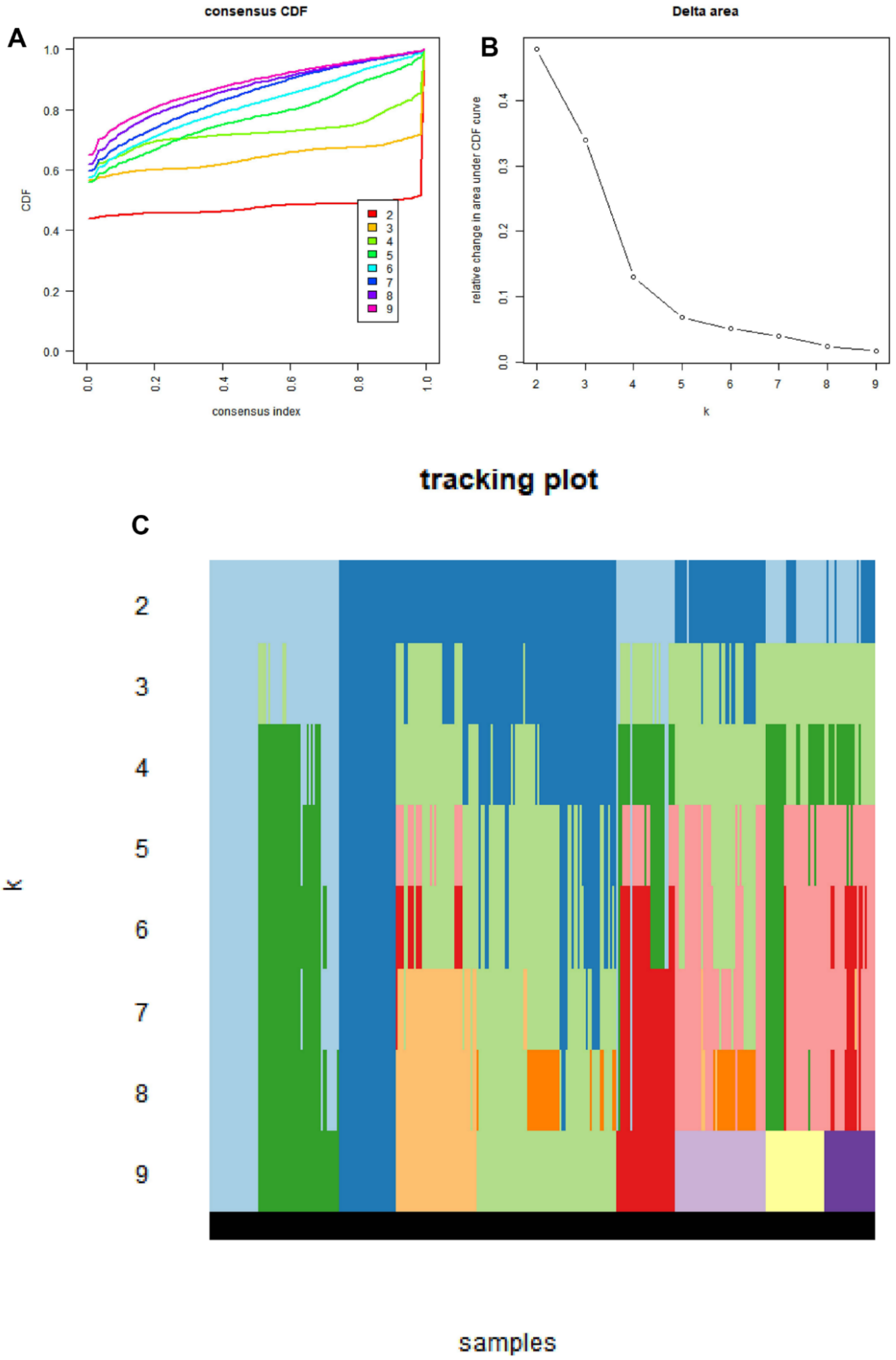

Supplementary Figure 4. Unsupervised cluster analysis to establish gene clusters. (**A**) CDF cumulative distribution curve. (**B**) Area under the CDF curve. (**C**) Tracking plot.

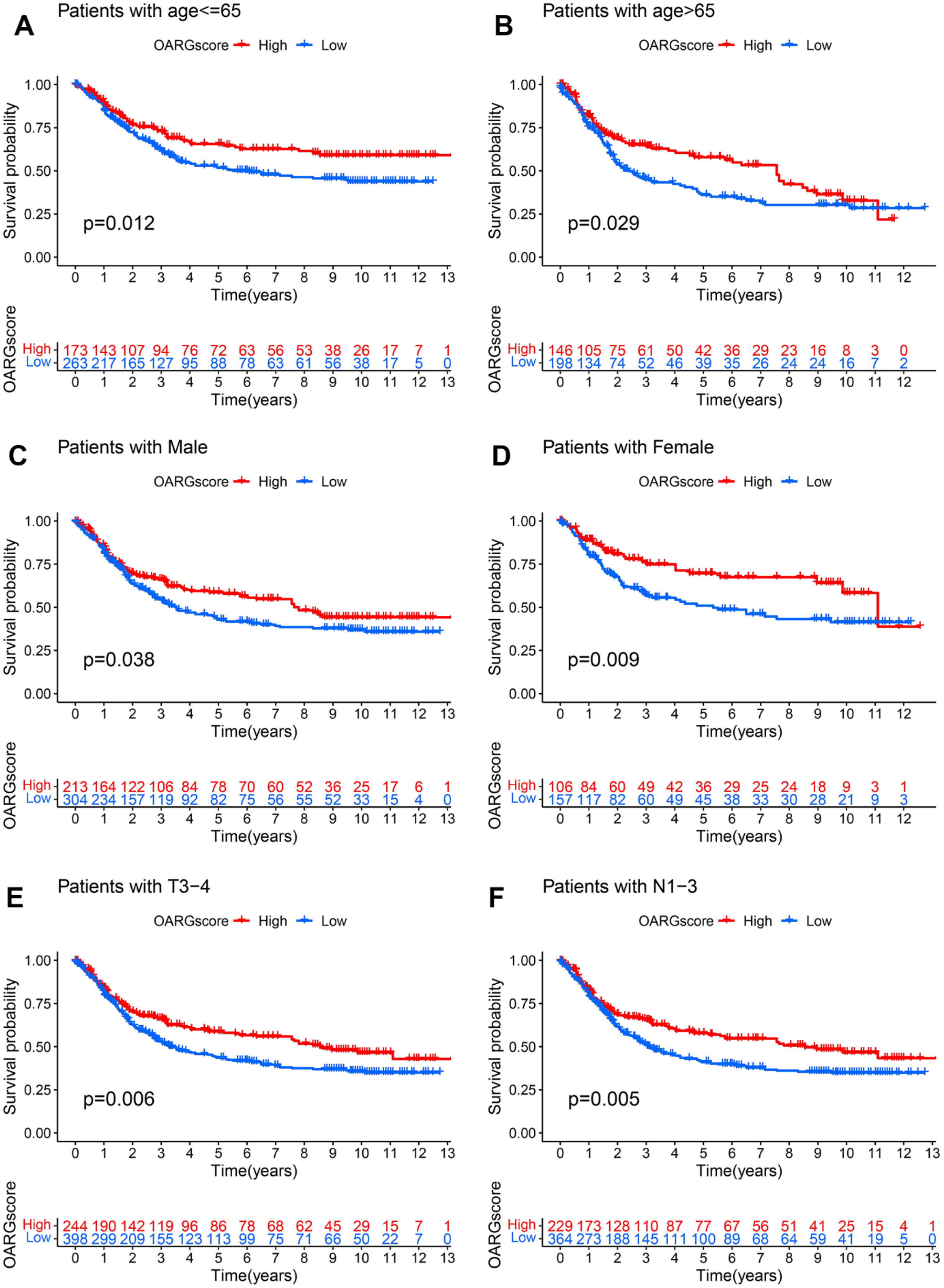

Supplementary Figure 5. Survival analysis between OARGscore and different types of patients. (**A**, **B**) Survival analysis between OARGscore and patients of age >/≤ 65. (**C**, **D**) Survival analysis between OARGscore and patients of different gender. (**E**) Survival analysis between OARGscore and patients with T3-4. (**F**) Survival analysis between OARGscore and patients with N1-3.
